# Gastroenteropancreatic Neuroendocrine Tumors: Does Tumor Location Affect Prognosis?

**DOI:** 10.34172/aim.33366

**Published:** 2025-01-01

**Authors:** Mehmet Sait Ozsoy, Cem Ilgin Erol, Muhammet Ali Aydemir, Hakan Baysal, Fatih Buyuker, Hatice Seneldir, Ozgur Ekinci, Tunc Eren, Orhan Alimoglu

**Affiliations:** ^1^Department of General Surgery, Faculty of Medicine, Istanbul Medeniyet University, Goztepe Prof. Dr. Suleyman Yalcin City Hospital, Istanbul, Turkey; ^2^Department of General Surgery, Erzurum City Hospital, Erzurum, Turkey; ^3^Department of Pathology, Faculty of Medicine, Istanbul Medeniyet University, Goztepe Prof. Dr. Suleyman Yalcin City Hospital, Istanbul, Turkey

**Keywords:** Digestive system, General surgery, Neuroendocrine tumors

## Abstract

**Background:**

Gastroenteropancreatic neuroendocrine tumors (GEP-NETs) are rare entities. Generally, they can be localized anywhere in the gastrointestinal or hepatobiliary tract. The purpose of our study is to evaluate the effect of tumor location on prognosis in patients with GEP-NET undergoing surgery. Our secondary objective is to examine other factors affecting the prognosis of patients with GEP-NET.

**Methods:**

We retrospectively analyzed data from 30 patients with GEP-NET who underwent surgery in the General Surgery Clinic between 2012 and 2022. The gNET group (n=18) included tumors located in the gastrointestinal tract, while the pNET group (n=12) included tumors located in the hepatopancreatobiliary system. Surgical, laboratory, radiological, and pathological findings of the patients, as well as follow-up outcomes were recorded and statistically analyzed.

**Results:**

In subgroup comparison, tumor size was found to be larger in the pNET group (*P*=0.002). The statistical analysis of recurrence (16.7% versus 33.3%) and mortality rates (16.7% versus 41.7%) between the subgroups (*P*=0.329 and *P*=0.210, respectively) did not reveal a significant difference. When all patients were evaluated, it was observed that advanced age, presence of carcinoma diagnosis, higher tumor grade, advanced TNM stage, larger tumor size, presence of lymphovascular or perineural invasion, elevated mitotic index, higher Ki-67 index, and having received adjuvant therapy increased the rates of recurrence and mortality.

**Conclusion:**

There was no statistically significant difference in survival outcomes between the GEP-NET groups located in the gastrointestinal tract and the hepatopancreatobiliary system.

## Introduction

 Gastroenteropancreatic neuroendocrine tumors (GEP-NETs) are a rare group of diseases.^1^ Reported on a series of 64 971 intrabdominal neuroendocrine tumors (iNETs), the annual age-adjusted incidence increased from 1.09 per 100 000 to 6.98 per 100 000 between 1973 and 2012.^2^ The most common locations of GEP-NETs are the small intestine (30.8%), rectum (26.3%), colon (17.6%), pancreas (12.1%), and appendix (5.7%).^3^

 In general, GEP-NETs can be localized anywhere in the gastrointestinal tract or hepatopancreatobiliary system, and the symptoms they cause are closely related to the location where the tumor develops. Since these tumors progress relatively slowly, they are more likely to be detected incidentally without causing symptoms.^1^ Gastrointestinal tract neuroendocrine tumors (gNETs) are often detected through non-specific findings in screening endoscopies and sometimes incidentally during the examination of appendectomy specimens.^4^ Nearly 40% of NETs located in the hepatopancreatobiliary system (pNETs) are detected incidentally.^5^ While pNETs are generally hormonally silent, they can produce various peptide hormones, including glucagon, insulin, and gastrin, potentially leading to clinical syndromes related to these hormones.^6^

 Gastrointestinal tract NETs take their origin from enterochromaffin cells, whereas pNETs are believed to arise from the islets of Langerhans. However, another hypothesis suggests that pNETs may originate from precursor cells in the ductal epithelium.^7^ Small intestine NETs have a relatively higher malignancy potential but tend to progress slowly in a metastatic setting. In contrast, gastric and rectal NETs generally exhibit a lower propensity for metastasis; however, they can progress rapidly once metastasis occurs.^4^

 The SEER database reports that at diagnosis, 53% of GEP-NET patients present with localized disease, 20% with local disease, and 27% with distant metastasis.^2^ Tumor grade is defined as follows: low-grade (G1) tumors have a mitotic index of 0‒1/2 mm^2^ or a Ki-67 proliferation index of 0‒2%; intermediate (G2) tumors have a mitotic index of 2‒20/2 mm^2^ or a Ki-67 proliferation of 3‒20%; and high-grade (G3) tumors show a mitotic index greater than 20/2 mm^2^ or a Ki-67 proliferation index exceeding 20%.^8^ The World Health Organization (WHO) classifies NETs as G1 and G2 which are considered well-differentiated, while G3 tumors are classified as poorly differentiated.^9^ Generally, early-stage GEP-NETs have a good prognosis, while tumor grade and location significantly influence the survival of patients with metastatic disease.^10^ Rectal NETs were reported have a better prognosis when compared to lesions in other locations, whereas pancreatic NETs had the worst prognosis.^11^

 In local or locoregional GEP-NETs, the preferred treatment is surgical resection with safe margins. For symptomatic pNETs larger than 2 cm, or for intermediate-high grade pNETs, a Whipple procedure or distal pancreatectomy should be performed depending on the location. Enucleation is another surgical option for these patients, but it poses higher risks regarding achieving safe surgical margins and lymph node dissection.^12^ Conversely, the treatment approach for low-grade, non-functioning, pNETs smaller than 2 cm is still debated. Some guidelines advocate for radical surgical resection of these tumors, while the European Neuroendocrine Tumor Society (ENETS) guidelines recommend surveillance for this patient group.^13^ In the management of gNETs, the most crucial determining factor is the tumor’s location. For jejunal and proximal ileal gNETs, segmental small bowel resections are performed. For tumors near or infiltrating the ileocecal valve, a right hemicolectomy is indicated.

 In about 25% of cases, multifocality is reported; therefore, during resections for gNETs, other bowel segments should be carefully inspected and palpated as means of formal surgical exploration. During these resections, including the mesentery is necessary for proper lymph node evaluation.^14^ As a general rule, for appendiceal NETs smaller than 1 cm, an appendectomy is considered sufficient. For tumors larger than 2 cm, a right hemicolectomy is recommended. For tumors of intermediate size (1‒2 cm) with significant mesoappendix invasion or those located at the base of the appendix, a right hemicolectomy should also be considered.^15^ For colonic gNETs, a formal colectomy should be performed.^16^ For rectal gNETs smaller than 2 cm, endoscopic or transanal resections can be planned. However, for larger tumors, abdominoperineal resection or low anterior resection should be performed with lymph node dissection.^17^

 In advanced tumors, medical therapy plays a crucial role in symptom control and prolonging survival in patients with locally advanced or metastatic GEP-NETs. Some somatostatin analogs, including octreotide and lanreotide, are commonly used as first-line treatments to alleviate symptoms related to hormone hypersecretion and to inhibit tumor growth by binding to somatostatin receptors on tumor cells.^18^

 The first aim of the present study is to evaluate the impact of tumor location on the prognosis of patients with GEP-NETs who underwent surgery. The secondary objective of the study is to investigate other factors influencing the prognosis of patients with GEP-NETs.

## Materials and Methods

**Figure 1:**
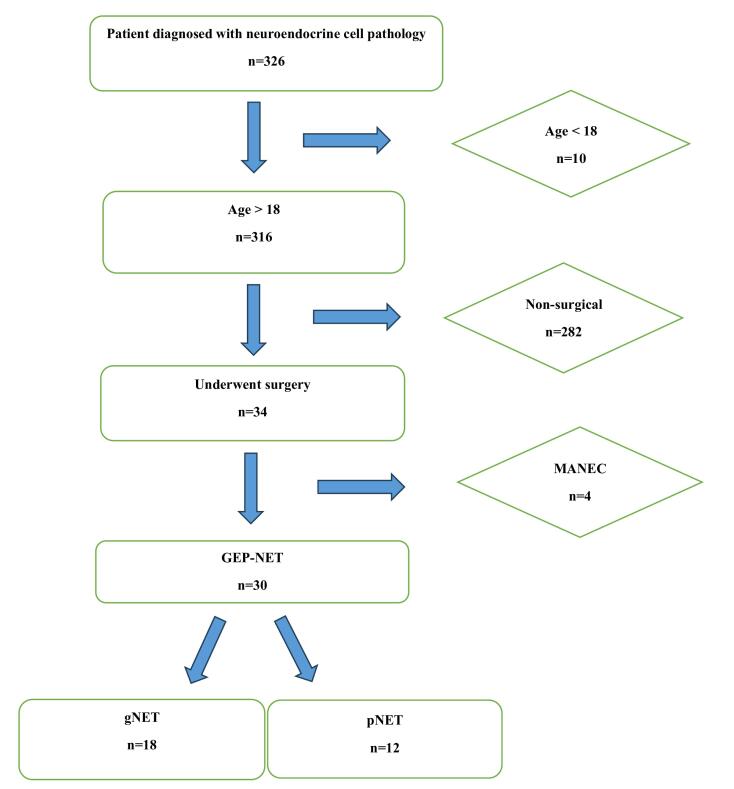
Patient Selection Flowsheet

 As shown in Figure 1, we retrospectively evaluated the historical records of 30 patients, who underwent surgery in the Department of General Surgery between 2012‒2022 and were reported as GEP-NET based on postoperative histopathological examinations. Data including intraoperative findings, early postoperative follow-ups, laboratory results, radiological imaging, and pathology reports were recorded. All patients were reached by phone and invited to regular follow-up visits at intervals of 6 months to the surgical oncological outpatient clinic as their clinical, laboratory and radiological and findings were documented simultaneously. Additionally, long-term follow-up results, including disease-free survival (DFS) and overall survival (OS) were recorded and evaluated.

 Patients were divided into two subgroups based on the tumor locations reported in the postoperative pathological examinations as the gNET group (tumors located along the gastrointestinal tract) and the pNET group (tumors located in the hepatopancreatobiliary system). Patients lost to follow-up, patients under 18 years of age, and those with incomplete data were excluded.

 All statistical analyses were conducted using the R version 3.6.1 (A language and environment for statistical computing. R Foundation for Statistical Computing, Vienna, Austria; https://www.R-project.org). Normality was assessed using the Shapiro-Wilk test and histograms. Depending on the normal distribution, either Student’s *t*-test or the Mann-Whitney U test was applied to compare continuous variables. Categorical variables were analyzed using Fisher’s exact test or Pearson’s chi-square test. Kaplan-Meier curves were generated for OS and DFS based on tumor location. A *P*-value of < 0.05 was considered statistically significant. Post hoc power analysis was conducted for the effect of location for recurrence and mortality using OpenEpi version 3.01 (https://www.openepi.com/).

## Results

 The study group consisted of 16 (53.3%) men and 14 (46.7%) women with a mean age of 51.7 ± 18.9 (median: 48, range: 19‒79) years. Among these patients, 53.3% (n = 16) had comorbidities, with hypertension being the most common (n = 7, 23.3%). A history of previous abdominal surgery was present in 10 (33.3%) patients. Postoperative histopathological examinations revealed that 24 (80%) of the patients had NET and 6 (20%) had NEC. Lymph node metastasis was observed in 10 (33.3%) patients, while distant metastasis was found in 3 (10%) patients. The appendix was the most common tumor location in 12 (40%) patients, followed by the pancreas in 9 (30%) patients, and the stomach in 2 (6.6%) patients (Table 1). After categorizing the patients into the gNET and pNET subgroups, it was found that 18 (60%) patients belonged to the gNET group and 12 (40%) to the pNET group. Three (10%) patients underwent reoperation post-surgery. The number of patients receiving adjuvant therapy was 8 (26.7%), and recurrence was observed in 7 (23.3%) patients. The mean OS was calculated as 54.7 ± 37.9 months, and the mean DFS was 52.5 ± 40.1 months. For cases identified as metastatic, the mean OS was 29.3 ± 25.7 (range: 13‒59) months.

**Table 1:** Demographics and Descriptive Parameters

**Parameter**			**N **(%)
		**Mean±SD**	
Age (y)		51.7 ± 18.9	30 (100.0)
		***n (%)***	
Gender	Men	16 (53.3)	30 (100.0)
	Women	14 (46.7)	
Comorbidity	Diabetes mellitus	4 (13.3)	16 (53.3)
	Hypertension	7 (23.3)	
	Coronary artery disease	2 (6.7)	
	Chronic obstructive pulmonary disease	1 (3.3)	
	Chronic kidney disease	1 (3.3)	
	Hyperlipidemia	3 (10.0)	
	Others	3 (10.0)	
Surgical history	Appendectomy	1 (3.3)	10 (33.3)
	Cholecystectomy	3 (10.0)	
	Colectomy	1 (3.3)	
	Herniorrhaphy	2 (6.7)	
	Cesarean section	1 (3.3)	
	Others	4 (13.3)	
Pathologic diagnosis	Neuroendocrine tumor	24 (80.0)	30 (100.0)
	Neuroendocrine carcinoma	6 (20.0)	
Tumor grade	1	13 (48.1)	27 (90.0)
	2	10 (37.0)	
	3	4 (14.8)	
AJCC stage	1	15 (50.0)	30 (100.0)
	2	5 (16.7)	
	3	7 (23.3)	
	4	3 (10.0)	
T stage	1	16 (53.3)	30 (100.0)
	2	3 (10.0)	
	3	9 (30.0)	
	4	2 (6.7)	
N stage	0	20 (66.7)	30 (100.0)
	1	7 (23.3)	
	2	3 (10.0)	
M stage	0	27 (90.0)	30 (100.0)
	1	3 (10.0)	
Tumor location	Ampulla vateri	1 (3.3)	30 (100.0)
	Appendix	12 (40.0)	
	Caecum	1 (3.3)	
	Pancreas	9 (30.0)	
	Jejunum	1 (3.3)	
	Liver	1 (3.3)	
	Colon	1 (3.3)	
	Stomach	2 (6.7)	
	Rectum	1 (3.3)	
	Gallbladder	1 (3.3)	
Tumor group	gNET	18 (60.0)	30 (100.0)
	pNET	12 (40.0)	
Multicentricity		3 (10.0)	30 (100.0)
Lymphovascular invasion		12 (40.0)	30 (100.0)
Perineural invasion		11 (36.7)	30 (100.0)
Synaptophysin		28 (100.0)	28 (93.3)
Chromogranin		27 (96.4)	28 (93.3)
Neural cell adhesion molecule 1 (CD56)		10 (83.3)	12 (40.0)
Reoperation history		3 (10.0)	30 (100.0)
Adjuvant therapy		8 (26.7)	30 (100.0)
Recurrence		7 (23.3)	30 (100.0)
Tumor size (cm)		2.4 ± 2.5	30 (100.0)
Mitosis rate (#)		5.73 ± 9.9	30 (100.0)
Ki-67 (%)		15.7 ± 28.0	30 (100.0)
Overall survival (months)		54.7 ± 37.9	30 (100.0)
Disease-free survival (months)		52.5 ± 40.1	30 (100.0)

N, Number of patients analyzed; n, Number of patients; SD, Standard deviation; gNET, Gastrointestinal neuroendocrine tumor; pNET, Hepatopancreatobiliary neuroendocrine tumor

 Statistical comparisons of the data from the two subgroups are presented in Table 2. No statistically significant differences were found between the gNET and pNET groups in terms of age, gender, comorbidities, previous surgeries, pathological diagnosis, tumor grade, TNM stages, multicentricity, or mitotic index. However, tumor diameter was significantly larger in the pNET group (*P* = 0.002). Additionally, no significant differences were observed in recurrence and mortality rates between the two subgroups (*P* = 0.329 and *P* = 0.210, respectively). Although the gNET group had more than double the median OS [61.1 (range: 9.7-110.0) months] and DFS [61.1 (range: 5.4‒110.0) months] compared to the pNET group (OS: 30.5 [1.7; 121.0] months, DFS: 25.8 [1.7; 121.0] months), this difference did not achieve statistical significance (*P* = 0.421 and *P*= 0.446, respectively).

**Table 2:** Comparison of the subgroups according to their demographic, pathologic, surgical and prognostic parameters

**Parameter**		**gNET (n=18) **	**pNET (n=12) **	***P***	**N (%)**
		**Median [min; max] **	**Median [min; max]**		
Age (years)		43.0 [19.0;77.0]	63.5 [36.0;79.0]	0.094	30 (100.0)
		** n (%) **	**n (%) **		
Gender	Women	8 (44.4)	6 (50.0)	1.000	30 (100.0)
	Men	10 (55.6)	6 (50.0)		
Comorbidity	Diabetes mellitus	2 (11.1)	2 (16.7)	1.000	30 (100.0)
	Hypertension	3 (16.7)	4 (33.3)	0.392	
	Coronary artery disease	1 (5.6)	1 (8.3)	1.000	
	Chronic obstructive pulmonary disease	1 (5.6)	0 (0.0)	1.000	
	Chronic kidney disease	0 (0.0)	1 (8.3)	0.400	
	Hyperlipidemia	1 (5.6)	2 (16.7)	0.548	
	Others	2 (11.1)	1 (8.3)	1.000	
Surgical history	Appendectomy	0 (0.0)	1 (8.3)	0.400	30 (100.0)
	Cholecystectomy	1 (5.6)	2 (16.7)	0.548	
	Colectomy	0 (0.0)	1 (8.3)	0.400	
	Herniorrhaphy	2 (11.1)	0 (0.0)	0.503	
	Cesarean section	1 (5.6)	0 (0.0)	1.000	
	Others	2 (11.1)	2 (16.7)	1.000	
Pathologic diagnosis	Neuroendocrine tumor	15 (83.3)	9 (75.0)	0.660	30 (100.0)
	Neuroendocrine carcinoma	3 (16.7)	3 (25.0)		
Tumor grade	1	9 (52.9)	4 (40.0)	0.762	27 (90.0)
	2	6 (35.3)	4 (40.0)		
	3	2 (11.8)	2 (20.0)		
AJCC stage	1	10 (55.6)	5 (41.7)	0.853	30 (100.0)
	2	3 (16.7)	2 (16.7)		
	3	3 (16.7)	4 (33.3)		
	4	2 (11.1)	1 (8.3)		
T stage	1	11 (61.1)	5 (41.7)	0.090	30 (100.0)
	2	0 (0.0)	3 (25.0)		
	3	5 (27.8)	4 (33.3)		
	4	2 (11.1)	0 (0.00)		
N stage	0	13 (72.2)	7 (58.3)	0.120	30 (100.0)
	1	2 (11.1)	5 (41.7)		
	2	3 (16.7)	0 (0.0)		
M stage	0	16 (88.9)	11 (91.7)	1.000	30 (100.0)
	1	2 (11.1)	1 (8.3)		
Multicentricity		3 (16.7)	0 (0.0)	0.255	30 (100.0)
Lymphovascular invasion		6 (33.3)	6 (50.0)	0.458	30 (100.0)
Perineural invasion		6 (33.3)	5 (41.7)	0.712	30 (100.0)
Synaptophysin		16 (100.0)	12 (100.0)	-	28 (93.3)
Chromogranin		16 (100.0)	11 (91.7)	0.429	28 (93.3)
Neural cell adhesion molecule 1 (CD56)		5 (83.3)	5 (83.3)	1.000	12 (40.0)
Reoperation history		3 (16.7%)	0 (0.00%)	0.255	30 (100.0)
Adjuvant therapy		5 (27.8%)	3 (25.0%)	1.000	30 (100.0)
Recurrence		3 (16.7%)	4 (33.3%)	0.392	30 (100.0)
Mortality rate		3 (16.7%)	5 (41.7%)	0.210	30 (100.0)
		**Median [min; max] **	**Median [min; max]**		
Tumor size *(cm)*		0.8 [0.3; 6.5]	2.8 [1.0; 11.0]	0.002	30 (100.0)
Mitosis rate (#)		1.0 [0.0; 30.0]	2.0 [0.0; 33.0]	0.294	30 (100.0)
Ki-67 (%)		3.0 [0.0; 90.0]	5.0 [1.0; 85.0]	0.150	30 (100.0)
Overall survival (months)		61.1 [9.7; 110.0]	30.5 [1.7; 121.0]	0.421	30 (100.0)
Disease-free survival (months)		61.1 [5.4; 110.0]	25.8 [1.7; 1210.]	0.446	30 (100.0)

N, Number of patients analyzed; n, Number of patients; Median [min; max], Median [minimum; maximum]; gNET, Gastrointestinal neuroendocrine tumor; pNET, Hepatopancreatobiliary neuroendocrine tumor.

 The statistical analyses to determine factors influencing prognosis, including recurrence and mortality, are provided in Tables 3 and 4. It was observed that advanced age, carcinoma diagnosis, higher tumor grade, advanced stages, larger tumor diameter, presence of lymphovascular or perineural invasion, increased mitotic index, higher Ki-67 values, and receiving adjuvant therapy were factors that increased both recurrence and mortality rates (*P* < 0.050). Tumor group was not found to be a significant factor for recurrence and mortality (*P* = 0.392 and *P* = 0.210, respectively). Notably, the presence of hypertension significantly increased mortality (*P* = 0.007). Additionally, the development of recurrence also increased mortality rates (*P* < 0.001).

**Table 3:** Comparison of the Parameters Affecting Recurrence

**Parameters**		**Recurrence**		***P***	**N **(%)
		**No (n=23)**	**Yes (n=7)**		
		**Median [min; max] **	**Median [min; max]**		
Age *(years)*		42.0 [19.0; 79.0]	75.0 [61.0; 78.0]	0.001	30 (100.0)
		***n (%)***	***n (%)***		
Gender	Women	10 (43.5)	4 (57.1)	0.675	30 (100.0)
	Men	13 (56.5)	3 (42.9)		
Comorbidity	Diabetes mellitus	2 (8.7)	2 (28.6)	0.007	30 (100.0)
	Hypertension	1 (4.3)	6 (85.7)		
	Coronary artery disease	2 (8.7)	0 (0.0)		
	Chronic obstructive pulmonary disease	0 (0.0)	1 (14.3)		
	Chronic kidney disease	1 (4.3)	0 (0.0)		
	Hyperlipidemia	1 (4.3)	2 (28.5)		
	Others	2 (8.7)	1 (14.3)		
Surgical history	Appendectomy	0 (0.0)	1 (14.3)	0.002	30 (100.0)
	Cholecystectomy	1 (4.3)	2 (28.6)		
	Colectomy	0 (0.0)	1 (14.3)		
	Herniorrhaphy	1 (4.3)	1 (14.3)		
	Cesarean section	1 (4.3)	0 (0.0)		
	Others	3 (13.0)	1 (14.3)		
Pathologic diagnosis	Neuroendocrine tumor	22 (95.7)	2 (28.6)	0.001	30 (100.0)
	Neuroendocrine carcinoma	1 (4.3)	5 (71.4)		
Tumor grade	1	13 (56.5)	0 (0.0)	< 0.001	27 (90.0)
	2	9 (39.1)	1 (14.3)		
	3	0 (0.0)	4 (57.1)		
	4	1 (4.3)	2 (28.6)		
AJCC stage	1	14 (60.9)	1 (14.2)	0.002	30 (100.0)
	2	5 (21.7)	0 (0.0)		
	3	4 (17.4)	3 (42.9)		
	4	0 (0.0)	3 (42.9)		
T stage	1	15 (65.2)	1 (14.2)	0.006	30 (100.0)
	2	1 (4.3)	2 (28.6)		
	3	7 (30.5)	2 (28.6)		
	4	0 (0.0)	2 (28.6)		
N stage	0	19 (82.7)	1 (14.2)	0.002	30 (100.0)
	1	3 (13.0)	4 (57.2)		
	2	1 (4.3)	2 (28.6)		
M stage	0	23 (100.0)	4 (57.1)	0.009	30 (100.0)
	1	0 (0.0)	3 (42.9)		
Tumor group	gNET	15 (65.2)	3 (42.9)	0.392	30 (100.0)
	pNET	8 (34.8)	4 (57.1)		
Multicentricity		3 (13.0)	0 (0.0)	1.000	30 (100.0)
Lymphovascular invasion		6 (26.0)	6 (85.7)	0.009	30 (100.0)
Perineural invasion		5 (21.7)	6 (85.7)	0.004	30 (100.0)
Synaptophysin		21 (91.3)	7 (100.0)	—	28 (93.3)
Chromogranin		21 (91.3)	6 (85.7)	0.250	28 (93.3)
Neural cell adhesion molecule 1 (CD56)		6 (26.0)	4 (57.1)	0.515	12 (40.0)
Reoperation history		3 (13.0)	0 (0.0)	1.000	30 (100.0)
Adjuvant therapy		2 (8.7)	6 (85.7)	< 0.001	30 (100.0)
Tumor size (cm)		0.9 [0.30; 7.00]	3.0 [1.00; 11.0]	0.020	30 (100.0)
Mitosis rate (#)		1.0 [0.00; 33.0]	16.0 [1.00; 32.0]	0.003	30 (100.0)
Ki-67 (%)		2.0 [0.00; 80.0]	25.0 [3.00; 90.0]	0.001	30 (100.0)

N, Number of patients analyzed; n, Number of patients; Median [min; max], Median [minimum; maximum]; gNET, Gastrointestinal neuroendocrine tumor; pNET, Hepatopancreatobiliary neuroendocrine tumor.

**Table 4:** Comparison of the Parameters Affecting Mortality

**Parameters**		**Mortality**		***P ***	**N **(%)
		**No (n=22)**	**Yes (n=8)**		
		**Median [min; max] **	**Median [min; max]**		
Age (years)		40.0 [19.0; 79.0]	72.5 [66.0; 78.0]	< 0.001	30 (100.0)
Gender	Women	9 (40.9)	5 (62.5)	0.417	30 (100.0)
	Men	13 (59.1)	3 (37.5)		
Comorbidity	Diabetes mellitus	2 (9.1)	2 (25.0)	0.003	30 (100.0)
	Hypertension	2 (9.1)	5 (62.5)		
	Coronary artery disease	1 (4.5)	1 (12.5)		
	Chronic obstructive pulmonary disease	0 (0.0)	1 (12.5)		
	Chronic kidney disease	0 (0.0)	1 (12.5)		
	Hyperlipidemia	2 (9.1)	1 (12.5)		
	Others	2 (9.1)	1 (12.5)		
Surgical history	Appendectomy	1 (4.5)	0 (0.0)	0.007	30 (100.0)
	Cholecystectomy	0 (0.0)	3 (37.5)		
	Colectomy	0 (0.0)	1 (12.5)		
	Herniorrhaphy	1 (4.5)	1 (12.5)		
	Cesarean section	1 (4.5)	0 (0.0)		
	Others	2 (9.1)	2 (25.0)		
Pathologic diagnosis	Neuroendocrine tumor	22 (100.0)	2 (25.0)	< 0.001	30 (100.0)
	Neuroendocrine carcinoma	0 (0.0)	6 (75.0)		
Tumor grade	1	12 (54.5)	1 (12.5)	0.016	27 (90.0)
	2	9 (41.0)	1 (12.5)		
	3	1 (4.5)	3 (37.5)		
AJCC stage	1	14 (64.6)	1 (12.5)	0.004	30 (100.0)
	2	4 (18.2)	1 (12.5)		
	3	4 (18.2)	3 (37.5)		
	4	0 (0.0)	3 (37.5)		
T stage	1	15 (68.1)	1 (12.5)	0.008	30 (100.0)
	2	2 (9.1)	1 (12.5)		
	3	5 (22.8)	4 (50.0)		
	4	0 (0.0)	2 (25.0)		
N stage	0	18 (81.8)	2 (25.0)	0.010	30 (100.0)
	1	3 (13.6)	4 (50.0)		
	2	1 (4.6)	2 (25.0)		
M stage	0	22 (100.0)	5 (62.5)	0.014	30 (100.0)
	1	0 (0.0)	3 (37.5)		
Tumor group	gNET	15 (68.1)	3 (37.5)	0.210	30 (100.0)
	pNET	7 (31.9)	5 (62.5)		
Multicentricity		3 (13.6)	0 (0.0)	0.545	30 (100.0)
Lymphovascular invasion		6 (27.2)	6 (75.0)	0.034	30 (100.0)
Perineural invasion		5 (22.8)	6 (75.0)	0.028	30 (100.0)
Synaptophysin		20 (90.9)	8 (100.0)	-	28 (93.3)
Chromogranin		20 (90.9)	7 (87.5)	0.286	28 (93.3)
Neural cell adhesion molecule 1 (CD56)		4 (18.2)	6 (75.0)	0.455	12 (40.0)
Reoperation history		3 (13.6)	0 (0.0)	0.545	30 (100.0)
Adjuvant therapy		3 (13.6)	5 (62.5)	0.016	30 (100.0)
Recurrence		1 (4.5)	6 (75.0)	< 0.001	30 (100.0)
Tumor size (cm)		0.9 [0.3; 7.0]	3.50 [1.0; 11.0]	0.006	30 (100.0)
Mitosis rate (#)		1.0 [0.0; 3.0]	18.0 [1.0; 33.0]	0.001	30 (100.0)
Ki-67 (%)		2.0 [0.0; 25.0]	52.5 [3.0; 90.0]	0.001	30 (100.0)

N, Number of patients analyzed; n, Number of patients; Median [min; max], Median [minimum; maximum]; gNET, Gastrointestinal neuroendocrine tumor; pNET, Hepatopancreatobiliary neuroendocrine tumor.

**Figure 2:**
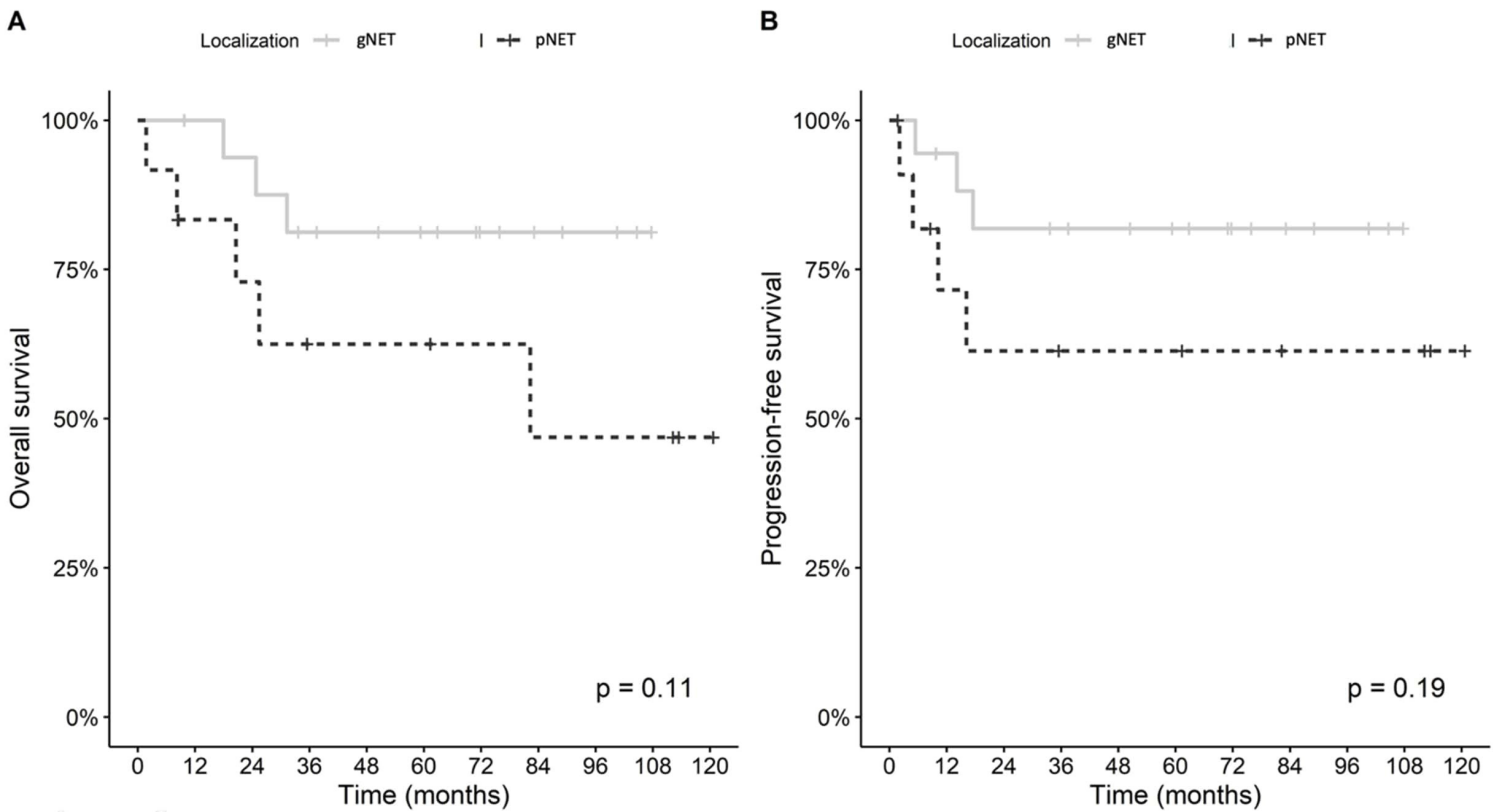
Comparison of Overall Survival (**A**) and Disease-Free Survival (**B**) between the Study Subgroups

 Kaplan-Meier survival curves evaluating OS and DFS based on tumor locations are shown in Figure 2. No significant differences were found in OS and DFS between the two subgroups (*P* = 0.110 and *P* = 0.190, respectively). Power analysis revealed that power was %17.8 for recurrence and %32.2 for mortality.

## Discussion

 Gastroenteropancreatic neuroendocrine tumors are malignancies that originate from neuroendocrine cells.^1^ The incidence of GEP-NETs is increasing worldwide, with most cases being NETs, some of which may progress slowly. However, highly proliferative tumors, such as G2/3 NETs and NECs characterized by rapid disease progression, are also reported.^19^ High-grade NETs are characterized by a high proliferation rate (Ki-67 > 20%) and include both well-differentiated G3 NETs and poorly differentiated NECs.^20^ While most cases generally involve NETs that typically progress slowly, rapid-progressing NECs can also be encountered in 10‒20% of cases.^8^

 In a study by Komiyama et al spanning 36 years with 43 patients, it was shown that the ratio of NET/NEC and the distribution of tumor stages at diagnosis differed according to the primary site. Patients who underwent surgical resection with G1 and G2 NETs had better prognoses, whereas those with NECs were associated with more advanced disease and poorer prognoses.^21^ A total of 20% of the patient group included in our study consisted of NEC cases.

 The clinical course of GEP-NETs can vary depending on primary tumor location.^22^ As a general rule, intestinal NETs, despite having relatively higher malignant potential, tend to progress slowly when metastatic, while gastric and rectal NETs tend to metastasize less frequently.^4,23^ The main treatment strategies for GEP-NETs include tumor resection, control of tumor growth and symptoms, and improving quality of life.^24^

 In a study examining factors affecting survival in GEP-NETs between 1975 and 2015, it was found that patients with pancreatic NETs had worse OS compared to others. It has been demonstrated that survival is shorter in patients over 60 years of age compared to those under 60 years, and in patients with G3 tumors compared to those with G1/G2 tumors.^25^ The multivariate analysis in the present study showed that male gender, tumor size greater than 2 cm, locoregional or metastatic disease, higher tumor grade, and higher TNM stage negatively impacted OS. Another result of the study is that tumors located in the appendix and small intestine have a more favorable impact on OS compared to other tumor locations.^25^ In our study, older age, higher tumor grade, advanced TNM stage, and greater tumor size were observed to negatively affect survival, while no significant difference in OS was found between gender groups. Furthermore, we did not observe a significant difference in OS between the gNET and pNET groups.

 A crucial parameter in the characterization of NETs is the histopathological diagnosis, which enables clinicians to ascertain the precise nature, grade, and metastatic potential of the tumors. For NETs, one of the prognostic factors is the tumor’s proliferative grade, which is determined by the percentage of tumor cells positive for Ki-67 immunostaining.^26^ Regarding the recurrence risk of GEP-NETs after curative surgical resection, a study by Merath et al in 2018 involving data from 1,477 patients reported that a high Ki-67 index, invasion of surrounding organs, lymph node positivity, and a tumor diameter greater than 3 cm increased the risk of recurrence.^27^ In our study, older age, presence of carcinoma diagnosis, higher tumor grade, advanced stages, large tumor diameter, presence of lymphovascular or perineural invasion, increased mitotic count, higher Ki-67 values, and receiving adjuvant therapy were found to increase recurrence rates.

 For patients with GEP-NETs, the Ki-67 proliferation index is an important biological marker. Although both WHO and ENETS use Ki-67 to determine prognostic groups, a definitive cutoff value has not yet been established.^28^ In our study, an increase in the Ki-67 value was found to increase both mortality and recurrence.

 In a study conducted by Chi et al, which examined the long-term outcomes of patients with GEP-NET, it was found that the 20-year disease-related survival rate for GEP-NET patients who underwent surgical resection was 77.5%. This rate dropped to around 50% for pancreatic tumors, while it was found to be 92.6% for rectal GEP-NET patients.^23,29^ Another study from the United States also examined the survival outcomes of GEP-NET patients and found that survival was shortest for patients with pancreatic tumors and longest for those with appendiceal and rectal tumors, with a statistically significant difference between the groups.^2^ In the current literature, not only is there a lack of extensive data on the outcomes of GEP-NET patients, but our literature review also revealed no similar grouping to our current study. Although a marked difference in OS and DFS was observed between our gNET and pNET groups, this difference did not reach statistical significance which may be due to the relatively small sample size of the present study.

 In a cohort study of 155 patients, Liu et al demonstrated that gastric NEC patients had a higher propensity for distant recurrences and a worse prognosis when compared to gastric adenocarcinoma and even poorly differentiated gastric adenocarcinoma.^30^ It has been proven that the prognosis of localized NETs is favorably consistent with a longer mean OS ( > 30 years) compared to metastatic NETs (mean OS: 12 months).^2,31^ In our study, the mean OS for the three cases (10%) found to be metastatic was 29.3 ± 25.7 (range: 13‒59) months, which was observed to be lower than the average OS of the total study group.

 The main limitations of our study are the single-institution context of the study, representing a relatively small sample size from a specific geographic region and its retrospective design. The power analysis indicated low statistical power for detecting effects in both recurrence (17.8%) and mortality (32.2%). However, considering the rarity of GEP-NETs, we believe that the data from the included patients provide valuable insights.

 In conclusion, our study on patients with rare GEP-NETs revealed that although there was a significant difference in survival outcomes between the two subgroups based on tumor locations (gastrointestinal tract and hepatopancreatobiliary system), this difference did not reach statistical significance. On the other hand, demographic, perioperative, and histopathological parameters of the patients had significant effects on recurrence and survival. We believe that prospective studies with larger patient groups will provide additional valuable data to the literature.
